# Thermal Stability and Sublimation of Two-Dimensional Co_9_Se_8_ Nanosheets for Ultrathin and Flexible Nanoelectronic Devices

**DOI:** 10.1021/acsanm.2c04640

**Published:** 2023-02-06

**Authors:** Dnyaneshwar S. Gavhane, Marijn A. van Huis

**Affiliations:** Soft Condensed Matter, Debye Institute for Nanomaterials Science, Utrecht University, Princetonplein 5, 3584 CC Utrecht, The Netherlands

**Keywords:** Co_9_Se_8_, 2D nanosheets, *in situ* transmission electron microscopy, thermal stability, sublimation

## Abstract

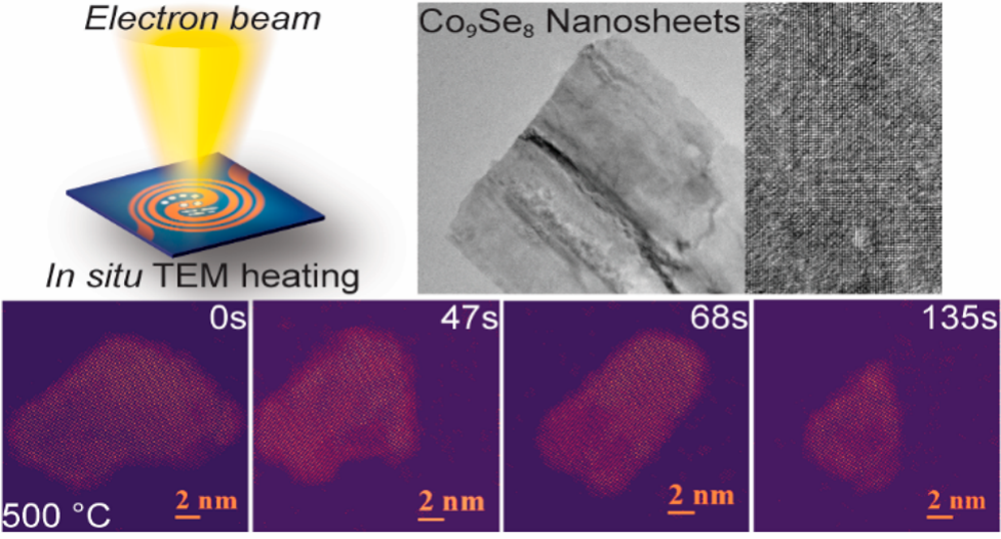

An understanding of the structural and compositional stability of nanomaterials is significant from both fundamental and technological points of view. Here, we investigate the thermal stability of half-unit-cell thick two-dimensional (2D) Co_9_Se_8_ nanosheets that are exceptionally interesting because of their half-metallic ferromagnetic properties. By employing *in situ* heating in the transmission electron microscope (TEM), we find that the nanosheets show good structural and chemical stability without changes to the cubic crystal structure until sublimation of the nanosheets starts at temperatures between 460 and 520 °C. The real-time observations of the sublimation process show preferential removal at {110} type crystal facets. From an analysis of sublimation rates at various temperatures, we find that the sublimation occurs through noncontinuous and punctuated mass loss at lower temperatures while the sublimation is continuous and uniform at higher temperatures. Our findings provide an understanding of the nanoscale structural and compositional stability of 2D Co_9_Se_8_ nanosheets, which is of importance for their reliable application and sustained performance as ultrathin and flexible nanoelectronic devices.

## Introduction

Atomically thin two-dimensional (2D) nanosheets have attracted great scientific and technological attention due to their excellent electronic and physical properties.^1−7^ In the context of applications, these 2D nanosheets play a vibrant role as basic building blocks for ultrathin, flexible, and transparent electronic devices, such as field-effect transistors, supercapacitors, electrodes, etc.^8−13^ Inspired by the exciting properties of these 2D nanomaterials, nanosheets of typically nonlayered materials have been synthetically prepared to expand the range of interesting 2D materials.^14−16^ This includes the synthesis of Co_9_Se_8_ ultrathin nanosheets^17^ that are 0.5 nm thin, which corresponds to half a unit cell of the bulk crystal structure. This reduction in dimension of bulk Co_9_Se_8_ to thin nanosheets (NSs) triggers scientific interest as novel thickness-dependent properties are generated in this way, which has led to numerous applications including ultrathin and flexible devices,^17^ catalysts for O_2_ eletroreduction,^18^ counter electrode materials for dye-sensitized solar cells,^19^ an alternative for Li-ion batteries for an energy storage system,^20^ a theranostic platform for biomedical applications,^21^ high capacity anode materials for lithium-ion batteries,^22^ etc. Co_9_Se_8_ quantum dots have also been used as a flexible resistive switching memory device for flexible and high-performance memory applications.^23^ In a recent density functional theory (DFT) study, it was found that the Co_9_Se_8_ nanosheets are ferrimagnetic with semimetallic features. The electronic band structure suggests that the nanosheets could be employed as a switchable half-metal where either the spin-up or the spin-down electrons can be made conductive.^24^

To reveal the physical and chemical properties of different nanomaterials, *in situ* transmission electron microscopy (TEM) techniques allow for following in real-time processes in chemical or solid reactions,^25−27^ structural/phase transformations,^28−31^ growth of nanomaterials,^32,33^ and the effects of external stimuli^34,35^ on the material. Several studies have been devoted to the thermal stability and sublimation processes of nanoparticles,^36−38^ 1D nanorods,^39^ and 2D nanosheets^38,40−42^ with real-time imaging using *in situ* heating in the TEM. Thermally induced sublimation and its accompanying processes are of fundamental importance and are of significant value to technological development. Close observation of this solid–vapor phase transition where atoms from the crystal lattice dissolve away into the gas phase can help to investigate the underlying mechanism at the atomic scale and illuminate the inverse process of crystal growth. Observing the sublimation process in 2D nanosheets is ideal because of their thickness, which makes them very suitable for imaging in projection in the electron microscope.^38,41,42^ As Co_9_Se_8_ nanosheets with half-metallic ferromagnetism could be used as ultrathin and flexible devices,^17^ in the technological applications, these nanosheets have to be compositionally and structurally stable at service temperatures. Most of the properties of nanomaterials are dependent on morphology, structure, and chemical composition, which are temperature sensitive. Annealing, thermal deposition of metal contacts, etc.,^43^ are typical thermal treatments involved in the incorporation of nanomaterials in devices. The study of chemical and structural stability of Co_9_Se_8_ nanosheets at elevated temperatures is therefore of high importance both from academic and industrial perspectives for ultrathin and flexible nanoelectronic devices.

In this work, we have employed *in situ* TEM heating to observe thermal stability and sublimation processes in hydrothermally synthesized Co_9_Se_8_ nanosheets.^17^*In situ* heating experiments were carried out in a Talos F200X microscope operated at 200 kV at a pressure of 10^–7^ Torr. The Co_9_Se_8_ NSs were characterized by high-resolution TEM (HR-TEM), scanning TEM (STEM), and energy dispersive X-ray (EDX) spectroscopy. More details on TEM characterization along with X-ray diffraction (XRD) and videos recorded during the sublimation process are presented in the Supporting Information.

## Experimental Section

### Synthesis of Co_9_Se_8_ Nanosheets

**Scheme 1:**
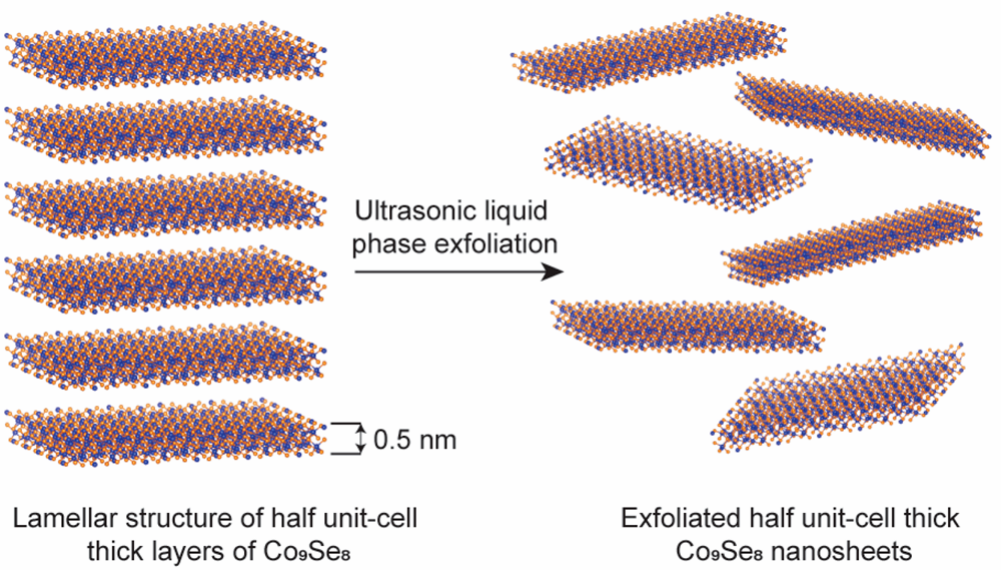
Schematic Illustration of Exfoliation of Half Unit-Cell Thick Co_9_Se_8_ Nanosheets

The cubic Co_9_Se_8_ NSs are synthesized as described in the existing literature.^17^ As-prepared samples were further exfoliated into fairly thin nanosheets using a bath sonicator as illustrated in Scheme 1. In this, 10 mg of Co_9_Se_8_ was dispersed in 20 mL of ethanol, and then, the mixture was ultrasonicated for 1 h. After ultrasonic treatment, the resultant dispersions were centrifuged at 3000 rpm for 10 min to remove the unexfoliated component.

### Methods: *In Situ* TEM

*In situ* transmission electron microscopy study of sublimation of Co_9_Se_8_ nanosheets was performed on a Talos F200X microscope operating with an acceleration voltage of 200 kV. Co_9_Se_8_ samples for *in situ* heating experiments were prepared by drop casting them directly onto MEMS chips with SiN_*x*_ membranes, which were mounted on a DENS solutions heating holder. The temperature was increased from room temperature to 500 °C in steps of 20 °C. Movies and images are recorded in real-time for observing the dynamic sublimation process.

## Results and Discussion

**Figure 1:**
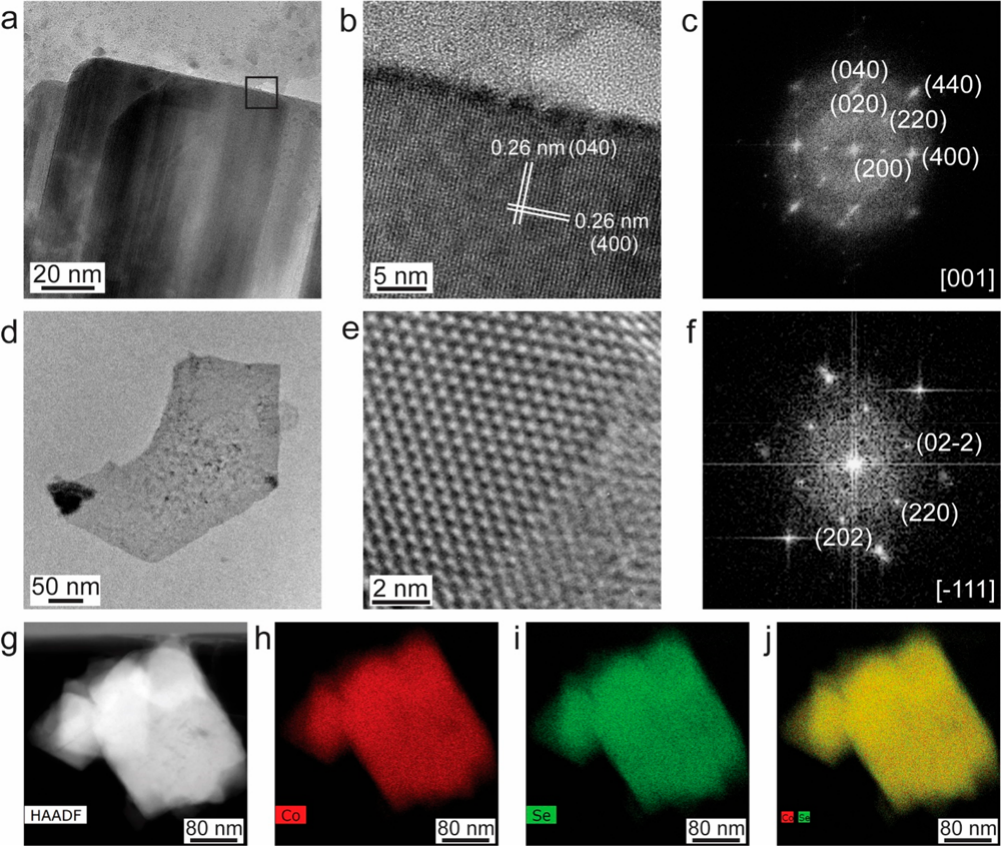
As-synthesized few-layer Co_9_Se_8_ NSs. (a and d) Low-magnification TEM images of few-layer thin Co_9_Se_8_ NSs. (b and e) HR-TEM images of the Co_9_Se_8_ lattice structure in its [001] and [-111] zone axis, respectively. (c and f) FFT patterns of Co_9_Se_8_ NSs shown in panels (b and e). (g) HAADF-STEM image of few-layer thin Co_9_Se_8_ NSs. (h and i) EDS compositional maps of Co_9_Se_8_ NSs in panel (g), for Co (in red) and Se (in green). (j) Overlay maps of Co and Se.

Figure 1 shows transmission electron microscope (TEM) and high-resolution TEM (HR-TEM) images and energy dispersive X-ray spectroscopy (EDS) compositional maps of few-layer Co_9_Se_8_ nanosheets (NSs). Figure 1a shows a plan-view TEM image of an as-synthesized few-layer thin Co_9_Se_8_ NSs supported on the SiN_*x*_ membrane of the heating chip. This shows well-defined, free-standing, and thinner nanosheets having a larger lateral size. These are oriented in basal planes {001} parallel to the nanosheets face and the edges are terminated by {110} type facets. The HR-TEM image in Figure 1b illustrates the high crystalline quality of the Co_9_Se_8_ NSs. The interplanar spacing of 0.26 nm corresponds to {400} lattice fringes. Figure 1c shows the corresponding fast Fourier transform (FFT) pattern with [001] zone axis (ZA) and shows a cubic structure of Co_9_Se_8_. The X-ray diffraction (XRD) pattern also shows the cubic Co_9_Se_8_ structure with a lattice parameter of *a* = 10.43 Å (see Figure S1a and Text S1). Figure 1d depicts another example of Co_9_Se_8_ NSs oriented in a {111} projection with edges terminated by {110} type facets. Figure 1e shows the HR-TEM image of one of the areas from Figure 1d, and the corresponding FFT pattern is shown in Figure 1f with ZA [1̅11] and {110} type planes. The high angle annular dark field-scanning TEM (HAADF-STEM) image in Figure 1g shows a thickness-dependent contrast of Co_9_Se_8_ NSs. Figure 1h–j display EDS compositional maps of Co_9_Se_8_ for Co, Se, and an overlay of both Co and Se, showing that the NSs are composed of Co and Se without any detectable impurities, and the molar ratio for Co/Se is ∼9:7.6 after quantitative analysis of EDS spectrum (Figure S1b), which is close to the stoichiometric ratio of Co_9_Se_8_. HR-TEM images in Figure S2 along with Text S2 describe the thickness of the Co_9_Se_8_ nanosheet to be ∼0.5 nm, which is the half-unit cell of the parameter of the bulk Co_9_Se_8_ crystal structure.

**Figure 2:**
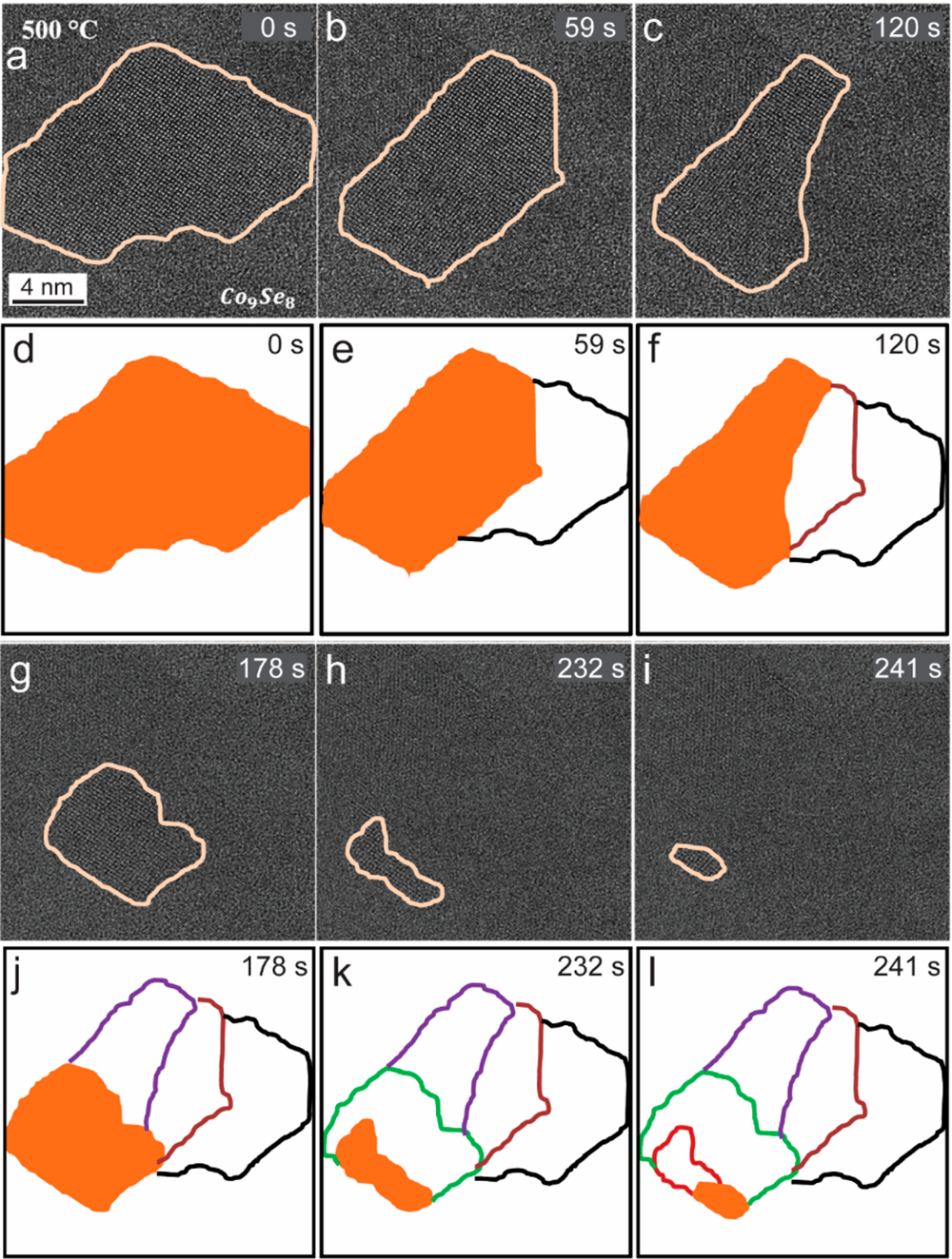
Structural evolution of Co_9_Se_8_ nanosheet after *in situ* TEM heating. (a) TEM image of the Co_9_Se_8_ nanosheet before the sublimation process started at 500 °C. (b, c, g, h, and i) TEM snapshots from the movie were recorded at 500 °C during the sublimation process at 59, 120, 178, 232, and 241 s, respectively. (d, e, f, j, k, and l) Qualitative image maps of the TEM images recorded at 500 °C, with contours of the initial nanosheet map at *t* = 0 s, and at other instants of time are shown by the differently colored dotted outlines; the contours of the nanosheets at the indicated times are shown by solid filled areas. All images are at the same scale.

Figure 2 shows the structural evolution of Co_9_Se_8_ NSs after *in situ* heating in the TEM at 500 °C. A very thin part of a nanosheet was monitored to record the sublimation process. The thin area is shown in Figure 2a and shows a cubic structure of Co_9_Se_8_ (see Figure S3 for HR-TEM analysis and Figure S4 for TEM simulated image). The presented TEM images in Figure 2 are the video frames (from Movie S1) captured at the indicated times. The heating treatment started by increasing the temperature from the room temperature with 20 °C increments. The Co_9_Se_8_ NSs showed good thermal stability until around 460 °C, after which the sublimation process took place where a mass loss was observed. Because, the bulk cubic Co_9_Se_8_ becomes unstable around 600 °C (Bohm et al., *Acta. Chem. Scand.*, 9, 1955), sublimation of Co_9_Se_8_ NSs was expected in this temperature range. As heating was done inside a TEM vacuum environment (10^–7^ Torr), and because nanomaterials are in general less stable than their bulk counterparts, the sublimation process was observed at a lower temperature. The sublimation temperature for solid material under a vacuum is typically lower than its melting temperature at atmospheric pressure.^41^ The time series shown in Figure 2 illustrates the sublimation in brief. Parts d–f and j–l Figure 2 are the qualitative image maps of the TEM images to visualize the sublimation process. An analysis of this process reveals that the sublimation takes place along different lattice plane directions. The area monitored in Figure 2 goes through a gradual sublimation that starts from the edges and progresses toward the inside, resulting in the sublimation of the complete nanosheet. During the sublimation, there was no noticeable change in the crystal structure of the NSs. We refer to this process as sublimation as no trace of solid material leftover was observed upon cooling the substrate, which would have yielded mass–thickness contrast in the TEM image. We have not observed any surface diffusion phenomena. Furthermore, we have also carried out STEM-EDS mapping for the elements before and after heating to the sublimation temperature, which is shown in Figure S5. Panels a–d in Figure S5 show images of TEM, HAADF-STEM, and elemental maps before the sublimation, while panel e shows an STEM-EDS elemental map after the sublimation of the nanosheet. In Figure S5, panels c and d show the elemental maps for Co and Se before the sublimation, confirming that the nanosheet is composed of Co and Se. After sublimation, we did not observe any Co and Se elements in the STEM-EDS maps (not shown). The Si from the SiN_*x*_ membrane and a trace of the shape of the nanosheet can still be seen on the membrane (due to minor contamination build-up around the nanosheet). Schematic model maps for corresponding filtered TEM images are shown in Figure S6 (see Text S3 for details on the preparation of schematic model maps) to analyze the process in detail and to show how the sublimation proceeds in different directions. Though the mass loss occurred along different facets, most of the sublimation takes place at (440) lattice planes as described in Figure S6. As shown in Figure S6, the mass loss starts at the (440) planes from the edge and progresses toward the interior of the NS. Further sublimation takes place through the detachment of atoms from (040) and (400) lattice planes, along with (440) planes until the whole NS sublimates.

The plot of the cumulative area sublimed against time in Figure S7 helps to understand the sublimation process quantitatively (details on the error bars are given in Text S4). The overall area of the NS under observation is ∼150 nm^2^, undergoing gradual sublimation in ∼4 min. As shown in Figure S7a, the increase in the cumulative area of the NS is slow for the first 50 s, where the sublimation begins from the edge of the NS and takes place at a steady rate along the (440) lattice plane direction. After the first 50 s, the sublimation occurred at a fast rate, where the mass loss took place along the directions of (040) and (400) lattice planes, along with sublimation in the direction of (440) lattice planes. The remainder of the area sublimed at a faster rate mostly along the (440) direction. Figure S7b,c shows the progression of sublimation in the direction of {400} lattice planes. Mass loss in these directions is small compared to that of in the direction of {440} type planes. The area sublimed along (400) is ∼10 nm^2^, whereas the area sublimed in the direction of (040) is ∼35 nm^2^. The sublimation along {440} planes covered most of the area of ∼100 nm^2^ of the NS and shows a steady rate of mass loss.

**Figure 3:**
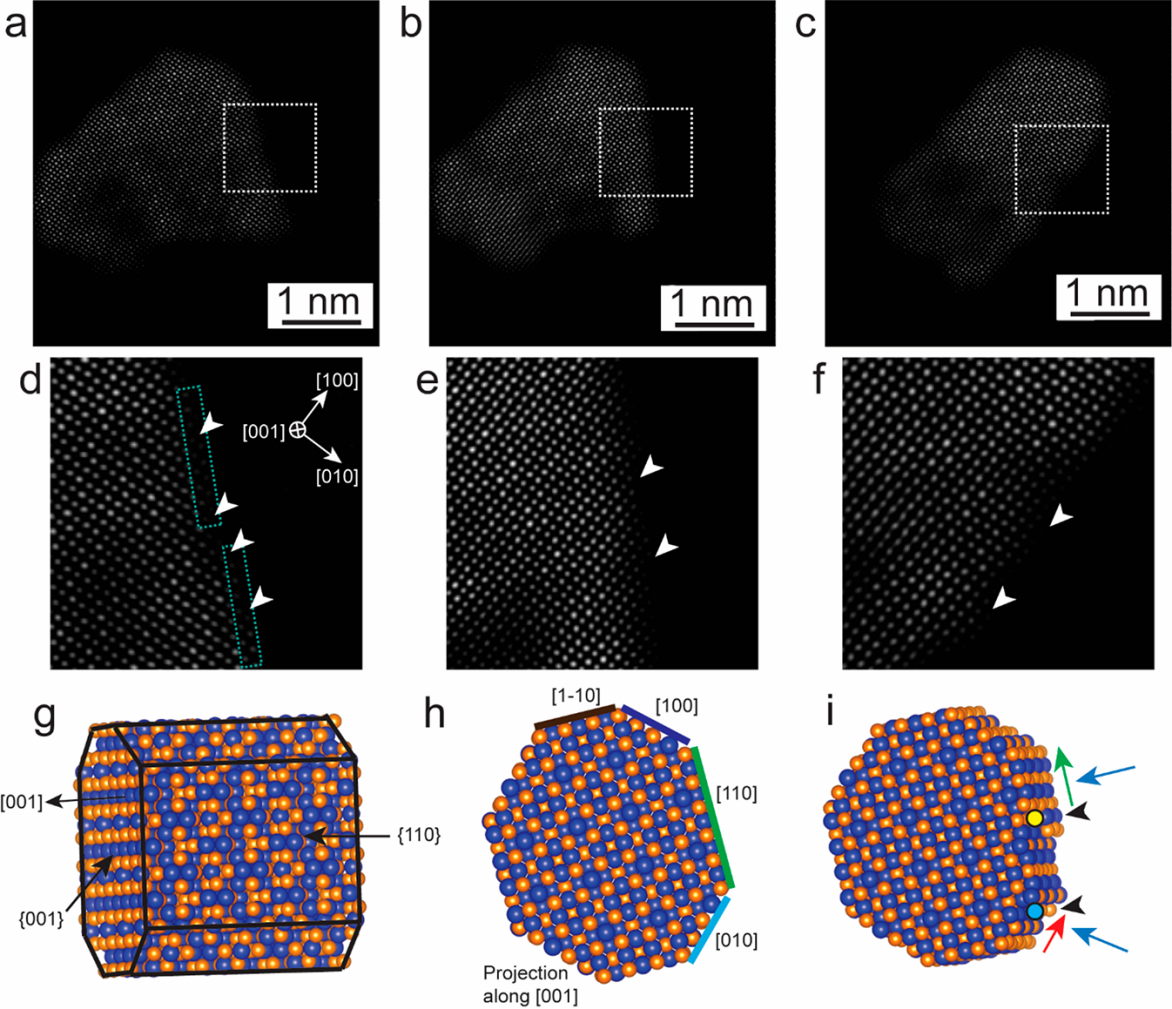
Atomic scale analysis of sublimation in Co_9_Se_8_ nanosheets. (a–f) Inverse FFT-filtered TEM images with background subtraction and contrast enhancement. (g–i) Schematic atomic models of a grain of Co_9_Se_8_ nanostructure.

It is challenging to say from the available high-resolution TEM images how the sublimation process proceeds at the atomic scale. To gain additional insights from the atomic scale information, we have filtered TEM images with inverse FFT along with background subtraction, contrast-enhancing, and intensity inversion. A few of the frames from Movie S1 are filtered in this fashion and are presented in Figure 3a, which shows the snapshot frame where the sublimation is propagating along the <110> direction. A close look at the sublimation propagation in Figure 3d suggests that steps are forming at the {110} facets marked with the white arrowheads and cyan-colored rectangles. Parts b and e of Figure 3 also show the steps at the {110} facets, marked with white arrowheads. A similar trend is observed when the sublimation propagation changes to <010> or <100> directions and is shown in Figure 3c,f, but in this case, the steps are forming at {010} and {100} facets, respectively.

The kinks are not visible while observing the sublimation process along [001] projection as shown in the Figure 3a–c. To resolve this better, the schematics of the grains of cubic Co_9_Se_8_ are shown in Figure 3g–i, with Figure 3g showing the crystal facets {110}, {001}, and edges along <001> direction while viewing along a slight off-axis <110> direction. Figure 3h shows schematically the edges of the grains while observing through the [001] projection. In Figure 3i, the yellow colored circle shows the kink formation at the {110} facet, creating the step at {110} facet shown by the black arrowhead. From the schematic, it is assumed that, during sublimation, the kinks propagate in <001> direction forming steps at the {110} facet and that step displacement along the <1-10> direction (shown by the green arrow) corresponds to sublimation along [110] direction (shown by the blue arrow). In case of the sublimation along [010] direction, the kinks form at the {010} facet (represented by the blue circle) and propagate in the <001> direction, creating a step on the {010} facet (shown by black arrowhead) whose movement is along the <100> direction (indicated by red arrow) while the sublimation occurs along the [010] direction (represented by the blue arrow). The measurement of the lateral step height of 0.18 nm corresponds to the distance of {110} lattice planes, and one set of {110} lattice planes is made of sublattices of both the atomic species Co and Se. Hence, the removal of one {110} plane involves the sublimation of both species. A similar trend is valid when the lateral step height of 0.26 nm corresponds to the {010} lattice planes and sublimation occurs in the [010] direction. It can be seen from the TEM images and the schematics that the kinks nucleate at the edge corners with two facets creating a crystal edge, and as discussed above, kinks propagate along the [001] direction and are only visible as a step while the whole atomic row along the [001] direction is sublimated.

It is likely that Co and Se species sublimate together since, in the case of separate Co and Se sublimation, a redox reaction would be needed to make these Co or Se atoms charge-neutral, while clusters of Co and Se can sublimate as (nearly) charge-neutral clusters without the need of adding or removing electrons. The movement of the kink is faster than the temporal resolution of the microscope, but the formation of single or double-step edges along the viewing direction [001] is visible in HR-TEM images.

**Figure 4:**
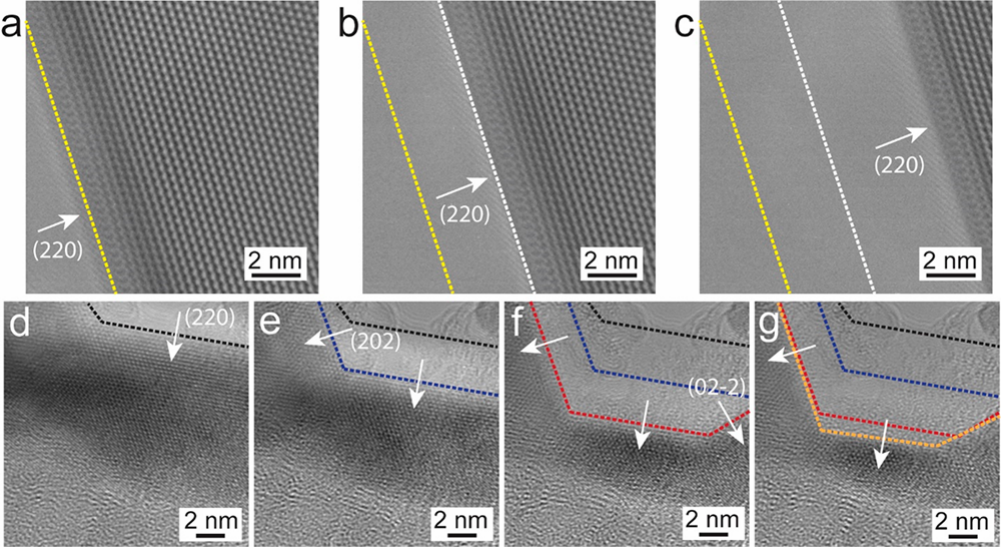
Sublimation of Co_9_Se_8_ NSs oriented in [111] ZA at 500 °C. (a) Fairly thin NS projected in [111] ZA with edges along (220) planes. (b and c) Time-series snapshots recorded from a sublimation process progresses from edges at (220) planes at different times. (d) Small part of NS before the sublimation begins. (e–g) Same part followed the sublimation along {220} planes at 500 °C.

In another experiment where the Co_9_Se_8_ NSs were oriented in the [111] ZA, the sublimation phenomenon was observed and is presented in Figure 4 (from Movie S2). As shown in Figure 4a, the NS is fairly thin and edges are terminated along (220) facets as indicated by the yellow dotted line. This is comparable to the NSs oriented in [001] ZA where the edges were also terminated along (440) planes. Increasing the temperature from room temperature to 500 °C in steps of 20 °C did not result in any change in the crystal structure of NSs, but at 500 °C, the NS started to sublimate gradually, starting from the edges along (220) planes (as shown in Figure 4b,c). The sublimation begins with a gradual detachment of atoms in the direction of (220) planes. The white dotted line in Figure 4b shows the progression of sublimation along (220) planes, and the emptied area between the yellow and white dotted lines depicts the sublimed area. Figure 4c shows how the sublimation proceeds along (220) planes without any mass loss along any of the other directions. Figure S8 shows the schematic model to show the progression of sublimation in NS at 500 °C. No mass loss occurred in any other direction than (220) suggests that these are the favorable directions for the mass loss and hence the sublimation. Parts d–g of Figure 4 show the sublimation of a part of the NS in the direction of {220} planes at 500 °C. In this case, sublimation progresses along {220} directions until the NS is fully sublimated. The dotted lines with blue, red, and orange colors represent the gradual progression of sublimation in the directions of {220} planes, whereas the black dotted line shows the initial position of the NS edge. In this process, the sublimation begins from one edge (top) and progresses toward the inner part, consuming the whole NS area and ending up at another edge (bottom). The bottom edge remained stable during the mass loss while sublimation progressed in the directions of various {220} planes from the top edge. Figure S9 shows a schematic model to depict the sublimation in NS projected in [111] ZA. A quantitative analysis of the sublimation observed in NSs projected in [111] ZA is presented in Figure S10. It can be seen from Figure S10a that the sublimed area increases gradually with time and the whole area of Figure 4a–c of ∼75 nm^2^ is sublimed within 25 s. As shown in Figure S10b the overall area of ∼90 nm^2^ of Figure 4d–g is sublimed within 50 s, where the sublimation occurred steadily for the first 30 s, after which it shoots up and consumed the whole area. In the second case (Movie S3), sublimation goes through different directions of {220} planes, and this might be the reason that the sublimation occurred much faster compared to the previous case. The fact that sublimation is preferred along the {220} planes in this temperature range is an indication that this surface is energetically less stable, as in general sublimation is favored to take place at less stable facets (as breaking up high-stability facets is energetically more costly). Unfortunately, (calculated) surface energies for the various facets are not available in the literature for comparison.

**Figure 5:**
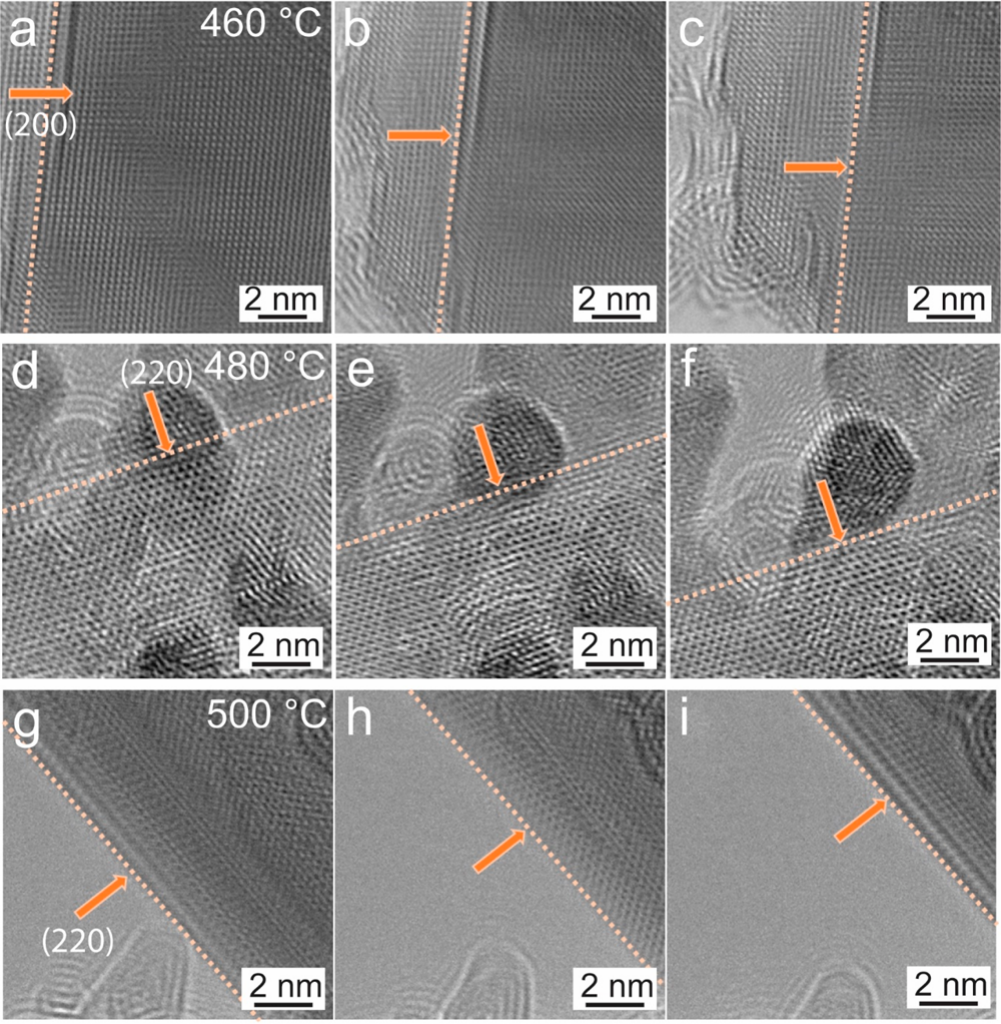
Sublimation process of Co_9_Se_8_ NSs at different temperatures. (a–c) Snapshots recorded from the punctuated and noncontinuous sublimation process along (200) planes at 460 °C. (d–f) Sublimation process of NSs projected in [111] ZA at 480 °C. (g–i) Continuous sublimation process along (220) crystal facets at 500 °C.

The sublimation of Co_9_Se_8_ NSs was studied at different temperatures to understand the phenomenon in detail, and observations of three sublimation events taking place at different temperatures are presented in Figure 5. As discussed above, sublimation was observed at 500 °C, which is continuous and steady in the direction of {110} type crystal facets. At lower temperatures, the mass loss occurred in a much different manner, as depicted in Figure 5a–c, the sublimation was observed at 460 °C. At this temperature, the sublimation occurred in a punctuated and noncontinuous way, where only a part of the NS was sublimed and the process stopped for a few seconds, after which it resumed again with other discrete sublimation events (see Movie S4). Figure S11a shows a plot of the cumulative sublimed area against time, for the noncontinuous sublimation. It can be seen that the sublimation is slow where ∼80 nm^2^ area is sublimed in ∼170 s and took place in an event-like manner along (200) crystal facets. At 480 °C, the process is close to the continuous sublimation where the mass loss occurred gradually until the whole area was consumed, as shown in Figure 5d–f (from Movie S5). The sublimation takes place along (220) crystal facets; the direction of progression is indicated by the arrows. Figure S11b shows quantitative measurements of the area of ∼90 nm^2^ that sublimed in ∼90 s and shows the continuity of the process. Continuous sublimation was observed in all experiments at 500 °C where the mass loss occurred gradually without any discrete events. One of the examples is shown in Figure 5g–i (from Movie S6) with NS oriented in [111] ZA, which sublimed along the (220) crystal facets shown by the arrows. The area of ∼160 nm^2^ sublimed in ∼90 s as shown in Figure S11c and depicts the continuous mass loss from the edge terminated along (220) crystal facets. Layer-by-layer sublimation was observed in a few-layered thin Co_9_Se_8_ NSs at 480 °C as shown in Figure S12 (from Movie S7). A layer on top of other layers sublimed first, and this continues until the entire NS has sublimed. This can be seen in Figure S12a–c where the edge of the top layer is indicated by the orange dotted line; it shows the progression in the sublimation; the noticeable difference in mass thickness contrast between images in a, b, and c represents the layer-by-layer sublimation. As shown in the plot in Figure S12d, this sublimation proceeds continually without any discrete mass loss events.

**Figure 6:**
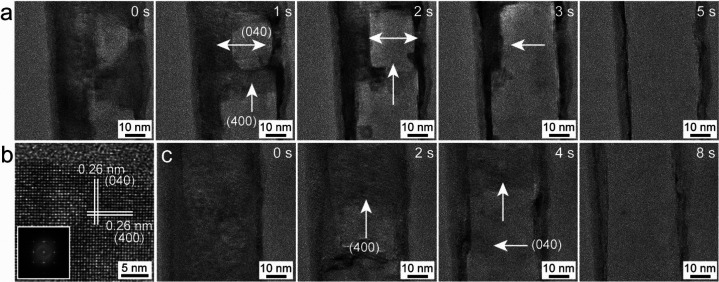
Sublimation process of Co_9_Se_8_ nanobelts. (a and c) Snapshots of the sublimation process from the Supporting movies (Movies S8 and S9), respectively. (b) HR-TEM image of the nanobelt with corresponding FFT in the inset.

In one of the experiments, with fairly thin nanobelts of Co_9_Se_8_, the sublimation at 520 °C occurs along the (400) and (040) crystal facets, as shown in Figure 6 and Movies S8 and S9. Figure 6a shows the snapshots captured at different times during the sublimation process and shows the progression of the mass loss in nanobelts along the {100} type crystal facets. It also shows that the entire area of the nanobelt in the field of view vanishes in ∼5 s, leaving the boundary most probably of the carbonous shell after the sublimation. The arrows in Figure 6a show the direction of progression of the sublimation and can be seen to be dominated by the {100} crystal facets. Figure 6c again shows another example of a similar type of nanobelt sublimating along {100} crystal facets at 520 °C, in less time (∼8 s). The HR-TEM image from one of the nanobelts is shown in Figure 6b along with the interplanar spacing of 0.26 nm (400) and the corresponding FFT in the inset. The irregularly shaped nanoplate of Co_9_Se_8_ shows the rapid sublimation that occurred at 520 °C in Figure S13 and Movie S10. The sublimation in this case is more stochastic and fast, which does not follow a particular direction for mass loss. These examples of sublimation in nicely shaped one-dimensional (1D) nanobelts and irregularly shaped nanoplates of Co_9_Se_8_ at 520 °C suggests that the morphology of these thin materials also plays a role in the directional mass loss. The confined nanobelts showed nicely directed mass loss along the {100} crystal facets, whereas the irregularly shaped nanoplate showed a fast and undirected mass loss.

## Conclusions

To summarize, thermal stability and sublimation of Co_9_Se_8_ NSs were investigated by real-time imaging at high temperatures by using *in situ* TEM. Co_9_Se_8_ NSs undergo sublimation under the vacuum environment in the TEM at temperatures between 460 and 520 °C. The observed sublimation is anisotropic and predominantly removes atoms from the least stable {110} type crystal facets. The observations of the filtered HRTEM images suggest step formation at {110} and {100}/{010}facets, which provide information on the kink formation and propagation that causes the sublimation to proceed through a kink-step-terrace type of sublimation at {110} and {100}/{010} crystal facets. Two different regimes were observed in the sublimation of NSs independent of thickness and size: noncontinuous and punctuated at lower sublimation temperatures and continuous and uniform at higher sublimation temperatures. Rapid and uncontrollable sublimation was observed after increasing temperature slightly above the temperature range of continuous sublimation. The present observations provide valuable insights into the sublimation process and this could be extended to other 2D nanomaterials having the same structures. These findings can play a vital role in the processability and operational range of technological applications of this material in nanosheet growth and the assembly and development of ultrathin and flexible nanoelectronic devices.

## Supporting Information Available

Figures of XRD and EDS analysis of Co_9_Se_8_ NSs, thickness of the 2D Co_9_Se_8_ nanosheets, HR-TEM images of the area under the observation, simulation of TEM image, EDS elemental analysis of before and after sublimation of Co_9_Se_8_ nanostructures, progression of sublimation along different lattice planes in Co_9_Se_8_ NSs, quantitative analysis of sublimation in Co_9_Se_8_ NS, sublimation of Co_9_Se_8_ NS oriented in [111] ZA, sublimation of NS in the directions of {220} planes, quantitative analysis of sublimation of Co_9_Se_8_ NS projected in [111] ZA, quantitative analysis of the sublimation process at different temperatures, layer-by-layer sublimation in a few-layered thick Co_9_Se_8_ NSs at 480 °C, and rapid sublimation in irregularly shaped Co_9_Se_8_ nanoplate at 520 °C (PDF)
Movie of structural evolution of Co_9_Se_8_ nanosheet after *in situ* TEM heating (sped up ×18, frame rate: 150 frames/s) (AVI)
Movie of sublimation of Co_9_Se_8_ NSs oriented in [111] ZA at 500 °C (sped up ×6, frame rate: 5 frames/s) (AVI)
Movie of sublimation of Co_9_Se_8_ NSs oriented in [111] ZA at 500 °C (sped up ×10, frame rate: 5 frames/s) (AVI)
Movie of noncontinuous and punctuating sublimation of Co_9_Se_8_ NSs at 460 °C (sped up ×18, frame rate: 25 frames/s) (AVI)
Movie of semicontinuous sublimation of Co_9_Se_8_ NSs at 480 °C (sped up ×5, frame rate: 10 frames/s) (AVI)
Movie of continuous and uniform sublimation of Co_9_Se_8_ NSs at 500 °C (sped up ×16, frame rate: 10 frames/s) (AVI)
Movie of layer-by-layer sublimation at 480 °C (sped up ×10, frame rate: 25 frames/s) (AVI)
Movie of sublimation in Co_9_Se_8_ nanobelts at 520 °C (real time, frame rate: 10 frames/s) (AVI)
Movie of sublimation in Co_9_Se_8_ nanobelts at 520 °C (real time, frame rate: 10 frames/s) (AVI)
Movie of rapid sublimation in Co_9_Se_8_ nanoplate at 520 °C (real time, frame rate: 10 frames/s) (AVI)

## Supplementary Material

an2c04640_si_001.pdf

an2c04640_si_002.avi

an2c04640_si_003.avi

an2c04640_si_004.avi

an2c04640_si_005.avi

an2c04640_si_006.avi

an2c04640_si_007.avi

an2c04640_si_008.avi

an2c04640_si_009.avi

an2c04640_si_010.avi

an2c04640_si_011.avi
