# Supplementary material for: Thermal Stability and Sublimation of Two-Dimensional Co9Se8 Nanosheets for Ultrathin and Flexible Nanoelectronic Devices
Source: ACS Appl Nano Mater. 2023 Feb 6;6(4):2421–8. doi: 10.1021/acsanm.2c04640 (PMC9972340; doi:10.1021/acsanm.2c04640)
Supplement: Supplementary file 1 — an2c04640_si_001.pdf [file an2c04640_si_001.pdf]

Supporting Information

Thermal Stability and Sublimation of Two-Dimensional Co_9Se_8 Nanosheets for Ultrathin and Flexible Nanoelectronic Devices

*Dnyaneshwar S. Gavhane, Marijn A. van Huis**

Dnyaneshwar S. Gavhane, Dr. Marijn A. van Huis
Soft Condensed Matter,
Debye Institute for Nanomaterials Science,
Utrecht University,
Princetonplein 5, 3584 CC, Utrecht,
The Netherlands

Corresponding author

*Email: m.a.vanhuis@uu.nl

Supplementary Text: 1

The X-ray diffraction pattern of the as-synthesized Co_9Se_8 nanosheets is shown in Figure S1(a). The XRD pattern shows peaks corresponding to (311), (222), (400), (511), (440), (531), and (622) lattice reflections, which corresponds to the cubic-Co_9Se_8 structure. The EDS spectrum of as-synthesized Co_9Se_8 is shown in Figure S1(b). The quantitative analysis of the EDS spectrum shows a ~ 9:7.6 ratio of Co and Se. The HRTEM image of a thin nanosheet of Co_9Se_8 under the observation is shown in Figure S3(a), which is imaged along the [001] zone axis. The interplanar spacing of 0.26 nm corresponds to the {400} type lattices. The FFT inverse filtered image is shown in Figure S3(b and c) for better visualization of the image in Figure S3(a).

Supplementary Text: 2

The thickness of the single-layer Co_9Se_8 nanosheets (NSs) measured from the high-resolution TEM images is found to be ~ 0.5 nm. TEM images of the folded edge of synthetic lamellar structures of the nanosheets (i.e., stacks of single-layer nanosheets) with a different number of layers show 0.5 nm thickness of the single-layer nanosheets, as indicated schematically in panel (c) of Figure S2 below. This thickness matches well with the thickness of the half-unit cell parameter (0.52 nm) of the bulk Co_9Se_8 crystal structure. The TEM images also show that the nanosheets with lamellar stacking vary from a single layer to several layers. The HRTEM image of a nanoplate (observed edge-on in Figure S2(b)b below) also suggests 0.5 nm thickness of the individual nanosheets. The TEM images indicate that the nanosheets (and nanoplates) are generally viewed along the [001] projection axis and that the typical lamellar stacking of the nanosheets is along the Z-axis.

Supplementary Text: 3

To quantify the sublimation from the TEM image, the 2D imaged area was considered as a reference to see the changes and taken to be representative of the loss of volume. The cumulative area in Figure S6 was analyzed using ImageJ image processing software by measuring the whole area first and then subtracting the remaining area of the next frame (frames chosen at the

indicated times in Figure 2 in the main text) after sublimation, etc. The number of frames was tracked to observe the sublimation along different lattice planes.

Supplementary Text: 4

The error bars in all the figures with graphs were calculated by taking into consideration the following aspects: the area was measured manually in all the cases at the times when significant changes were observed. This creates a part of error in the manual measurements. Secondly, the measurement error from the microscope as the microscope is not the aberration-corrected one which produces a part of the instrumental error in the measurement itself. To compensate for all the other errors in the measurements, a constant error of ±5 nm^2 is given to all the values in the graphs except the graphs in Figures S7 b and c where the error was considered to be ±1 and ±2 nm^2, respectively.

Supporting Figures

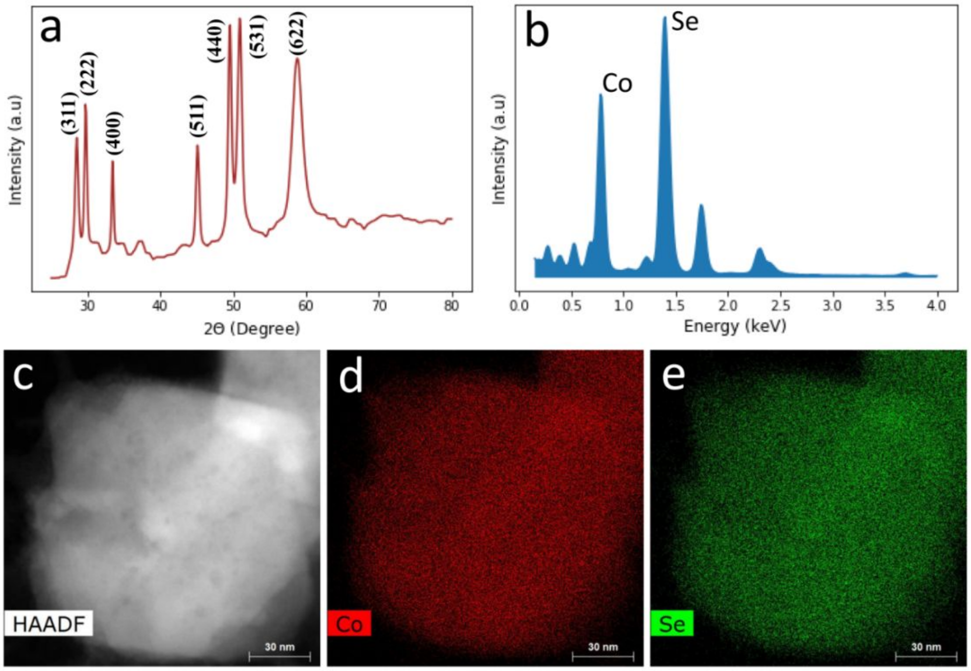

Figure S1. XRD and EDS analysis of Co_9Se_8 NSs. (a) XRD pattern of as-synthesized Co_9Se_8 nanosheets. (b) EDS spectrum of as-synthesized Co_9Se_8 recorded at room temperature. (c) HAADF-STEM image of Co_9Se_8 NSs. (d and e) EDS elemental maps for Co (red) and Se (green).

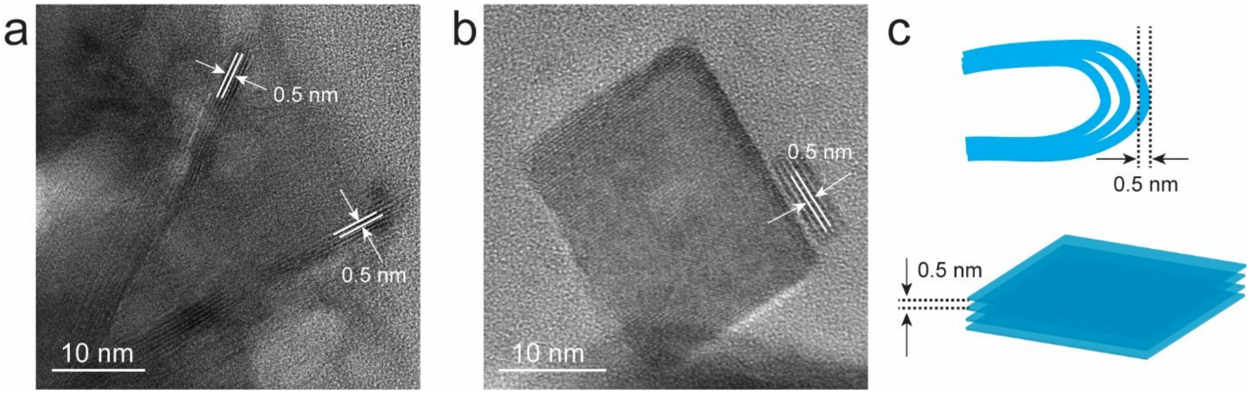

Figure S2. The thickness of the 2D Co_9Se_8 nanosheets. (a) Lamellar stacking of the 2D Co_9Se_8 nanosheets. (b) Thin horizontally and vertically oriented nanoplate of Co_9Se_8. (c) Schematic illustration of the folded edge of Co_9Se_8 nanosheets and basal plane oriented Co_9Se_8 nanosheets.

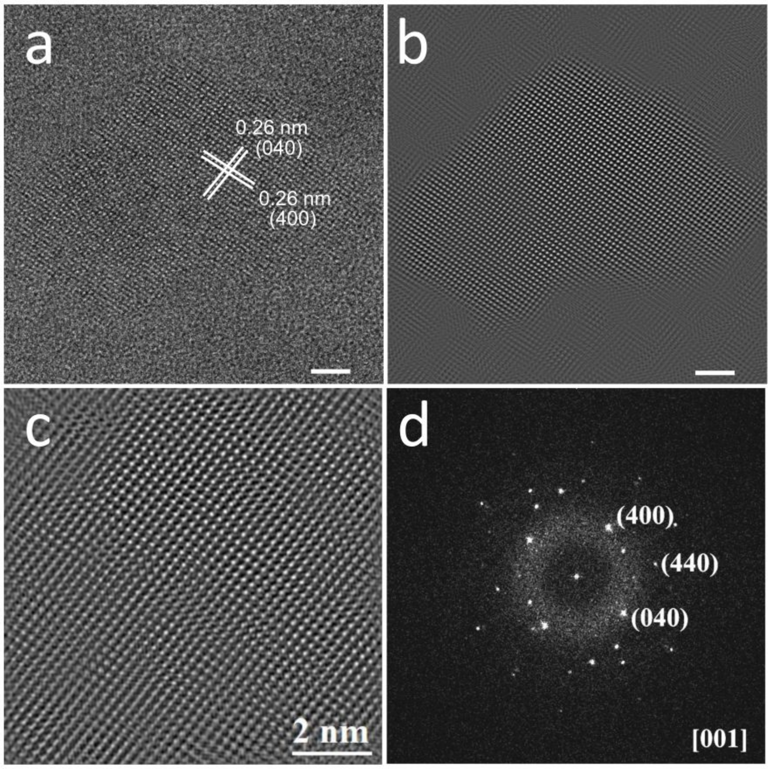

Figure S3. HR-TEM images of the area under observation. (a) A very thin area of Co_9Se_8 NS projected in [001] ZA, was chosen to observe the sublimation process. (b) Inverse FFT filtered the TEM image of the area in a. (c) Magnified image of the area in b, showing {400} type lattice fringes. (d) Corresponding FFT pattern illustrating the cubic structure of Co_9Se_8. The scale bar in (a) and (b) is 4 nm.

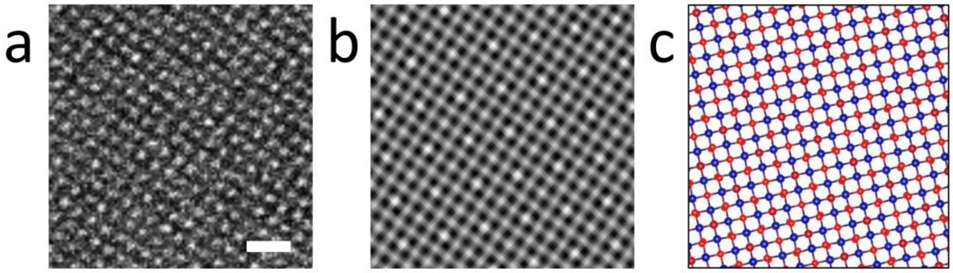

Figure S4. Comparison of experimental and simulated TEM images. (a) Enlarged view of the TEM image of Co_9Se_8 NSs. (b) Simulated TEM image. (c) Ball and stick model of Co_9Se_8 with blue balls representing cobalt atoms and red balls selenium atoms. The scale bar is 1 nm.

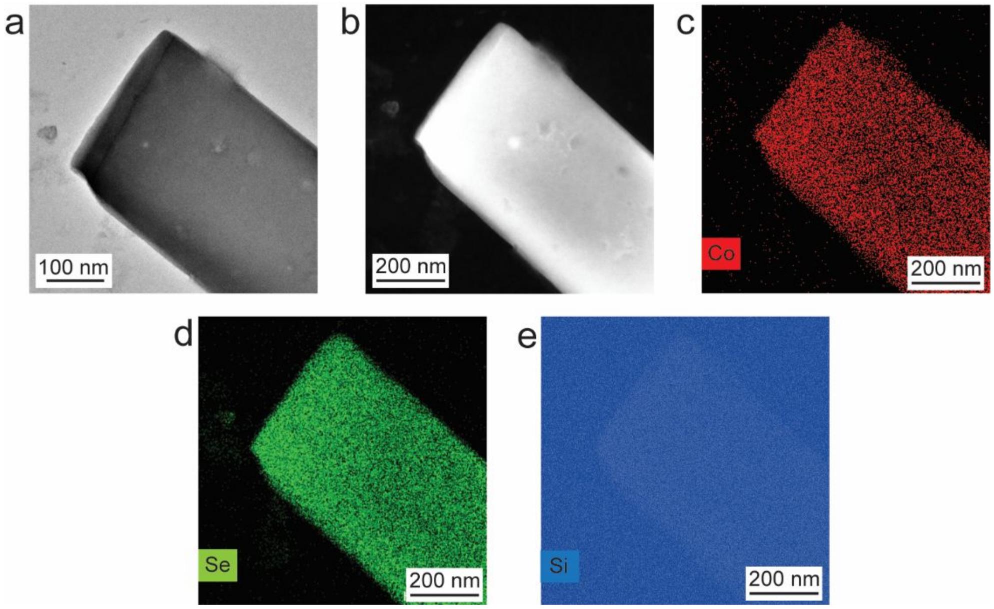

Figure S5. EDS elemental analysis of Before and after sublimation of Co_9Se_8 nanostructures. (a) TEM. (b) HAADF-STEM. (c and d) EDS elemental maps for Co and Se respectively before the sublimation. (e) EDS elemental map for Si from the SiN_x membrane with the trace of the shape of the Co_9Se_8 nanostructure after the sublimation. The elemental maps of Co and Se after sublimation did not have any counts (black) and are not shown.

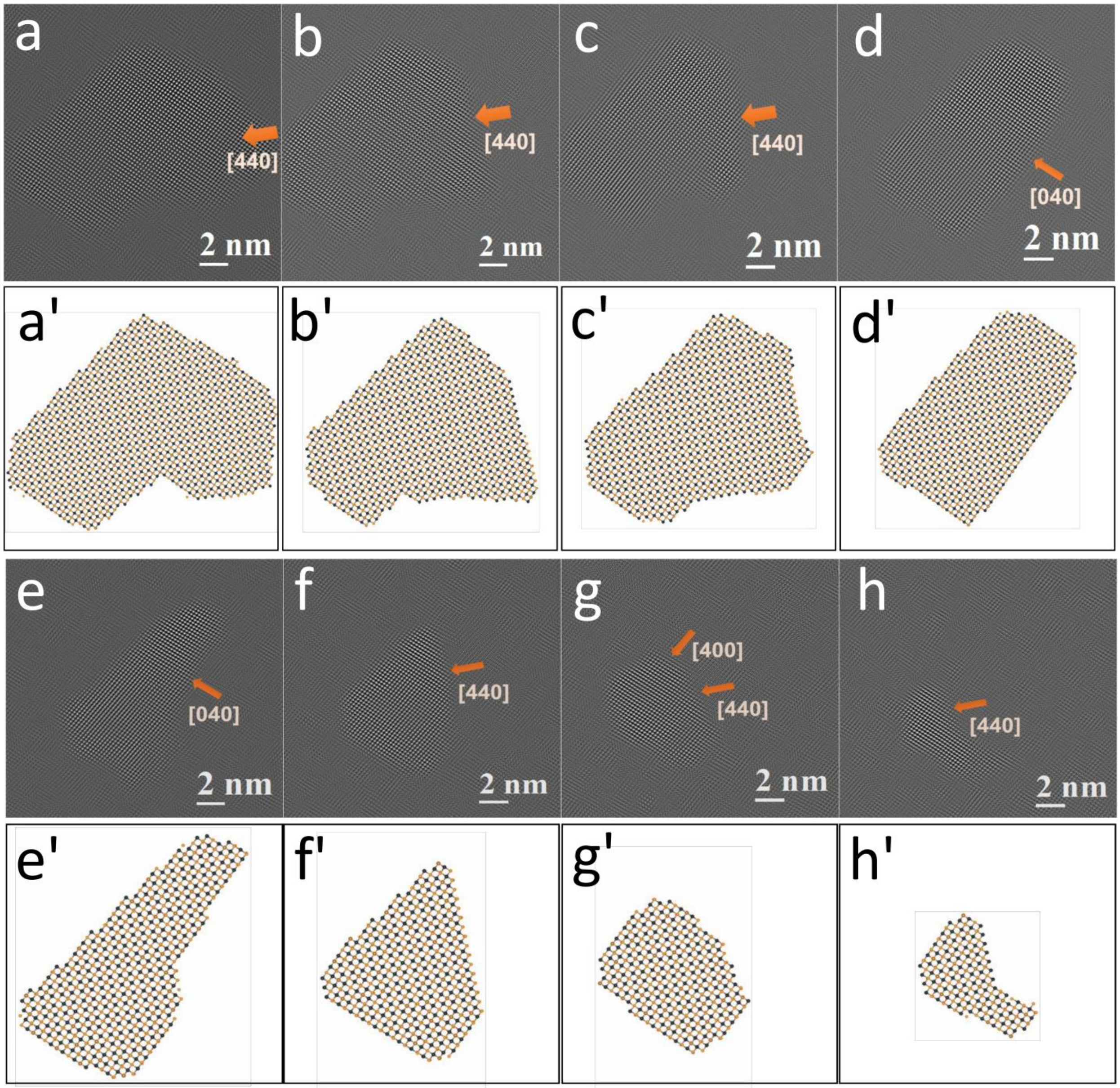

Figure S6. Progression of sublimation along different lattice planes in Co_9Se_8 nanosheets. (a-h) Inverse FFT filtered TEM time-series snapshots during the sublimation process. Arrows indicate the progression of sublimation in the direction of a particular lattice plane in each frame. (a'-h') Schematic maps of the TEM images to visualize the sublimation with ball and stick models, where black dots represent Cobalt and yellow balls occur indicate Selenium. The magnifications of the TEM images and the schematic maps are not the same.

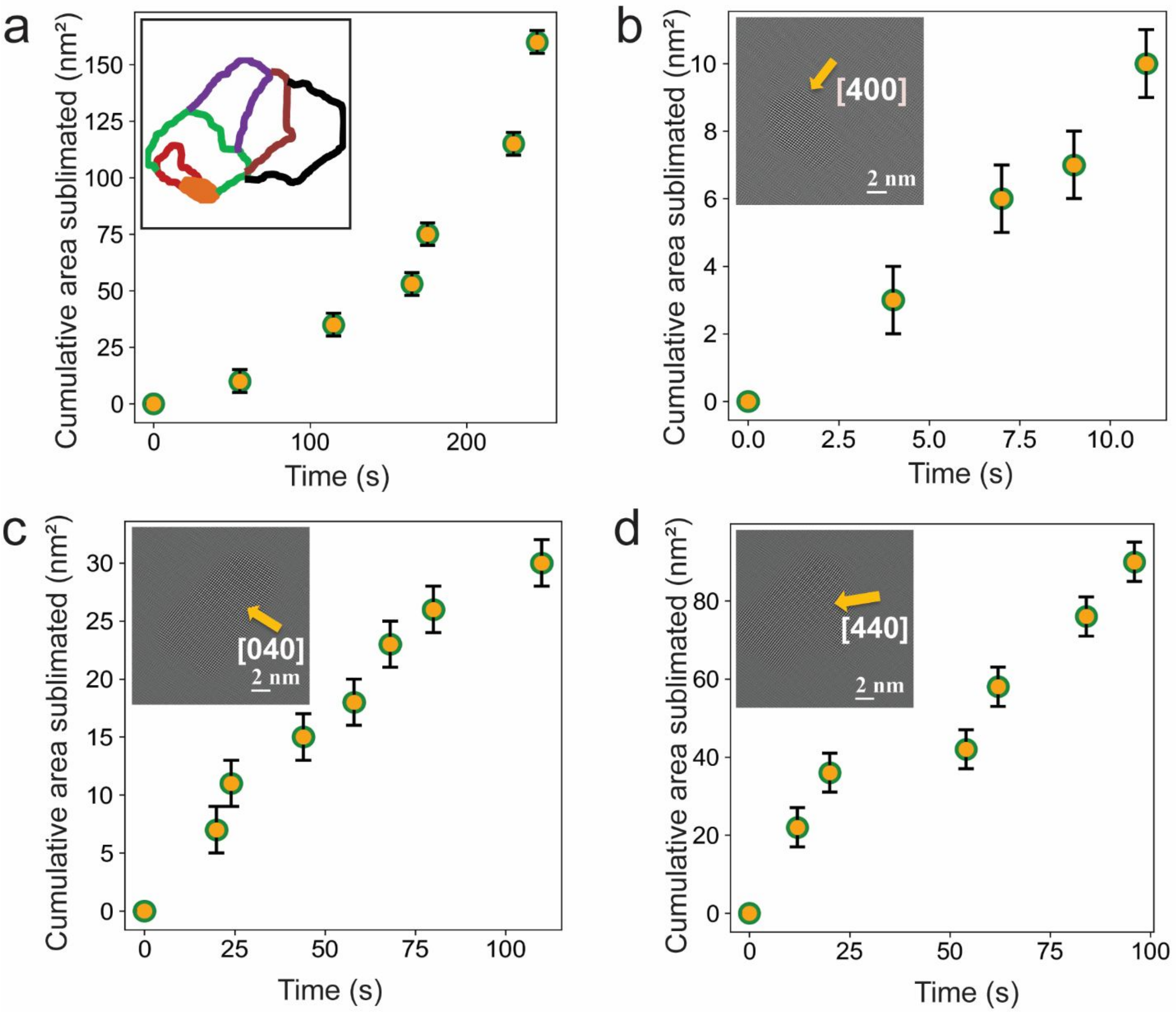

Figure S7. Quantitative analysis of sublimation in Co_9Se_8 nanosheets. (a) A plot of the cumulative sublimed area as a function of time. The inset shows the map of cumulative areas. (b and c) A cumulative sublimed area occurred in the direction of {400} lattice planes. (d) The plot of the area sublimed along {440} lattice planes as a function of time. Insets in b, c, and d show the inverse FFT TEM images of the frames and arrows show the direction of sublimation in those particular lattice planes. The scale bar for the images in the inset is 2 nm. (Details on the error bars are given in Supplementary Text 4)

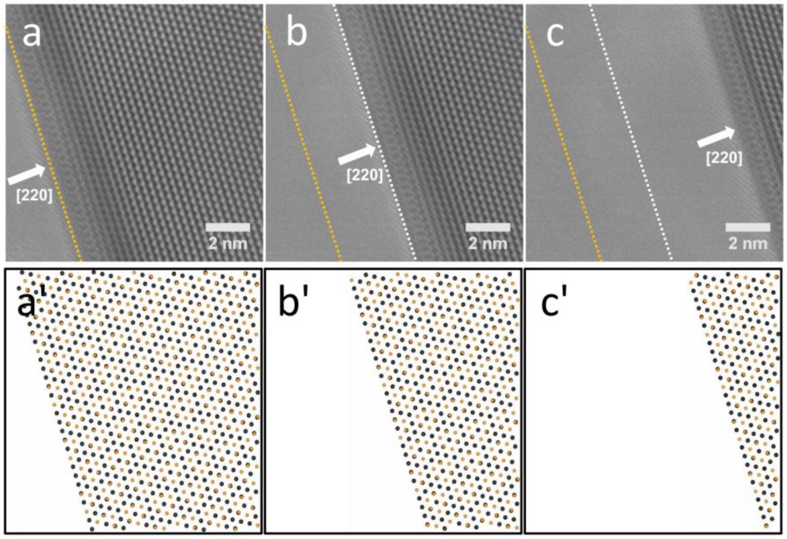

Figure S8. Sublimation of Co_9Se_8 NS oriented in [111] ZA. (a-c) TEM image snapshots were captured from the sublimation process along (220) planes. (a'-c') Schematic model showing the progression of sublimation along (220) planes with black and orange dots representing Cobalt and Selenium atoms respectively. The scales of TEM images and schematic models are not the same.

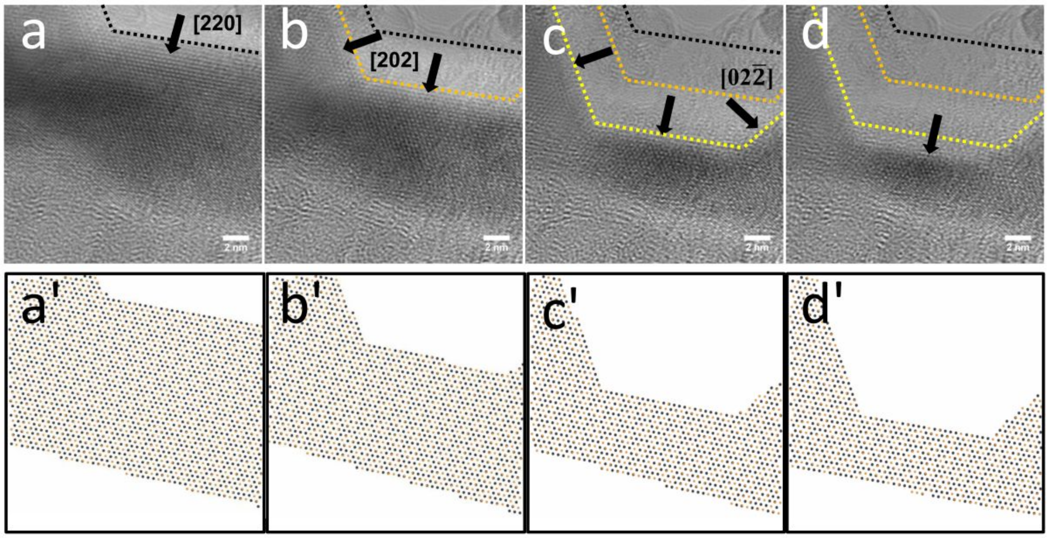

Figure S9. Sublimation of NS in the directions of {220} planes. (a-d) TEM image snapshots captured during the sublimation go through {220} planes. Dotted lines with orange and yellow colors show a progression of sublimation and the black dotted line represents the initial position of the edge from where sublimation began. Arrows indicate the direction of sublimation in each TEM image. (a'-d') Schematic model depicting the sublimation from TEM images with black and orange dots for Cobalt and Selenium respectively.

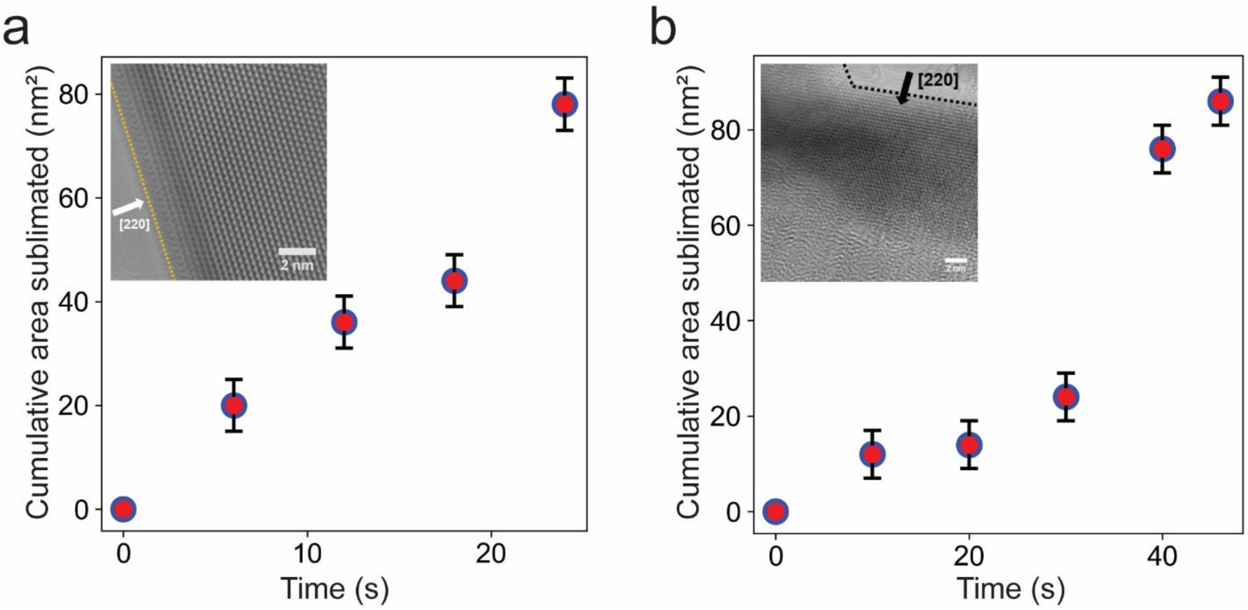

Figure S10. Quantitative analysis of sublimation of Co_9Se_8 NS projected in [111] ZA. (a and b) Measurement of area sublimed in the direction of (220) planes in NS within the indicated time, Edge terminated by (220) shown by orang colored dotted line (black colored dotted line in b) and an arrow showing the progression of sublimation. Insets in a and b show the TEM images before the sublimation progresses along the planes indicated by the arrows. (Details on the error bars are given in Supplementary Text 4)

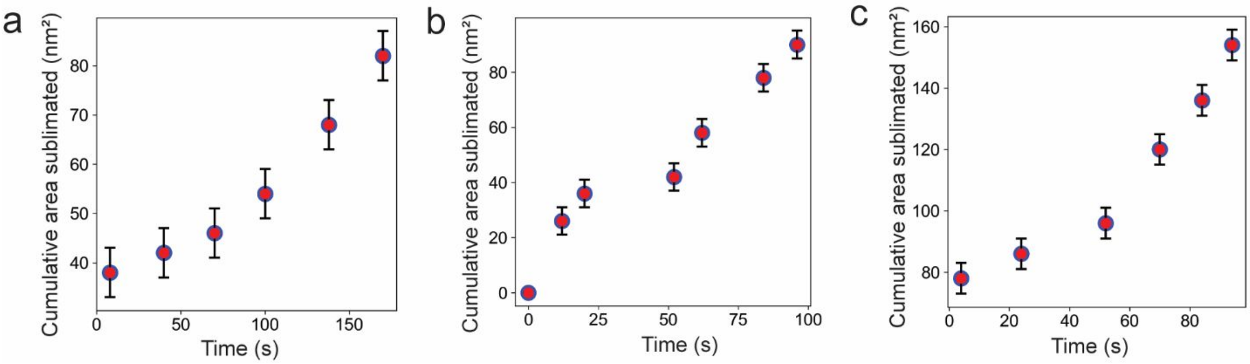

Figure S11. Quantitative analysis of the sublimation process at different temperatures. (a) Non-continuous sublimation at 460 °C. (b) Sublimation at 480 °C. (c) Continuous and steady sublimation at 500 °C. (Details on the error bars are given in Supplementary Text 4)

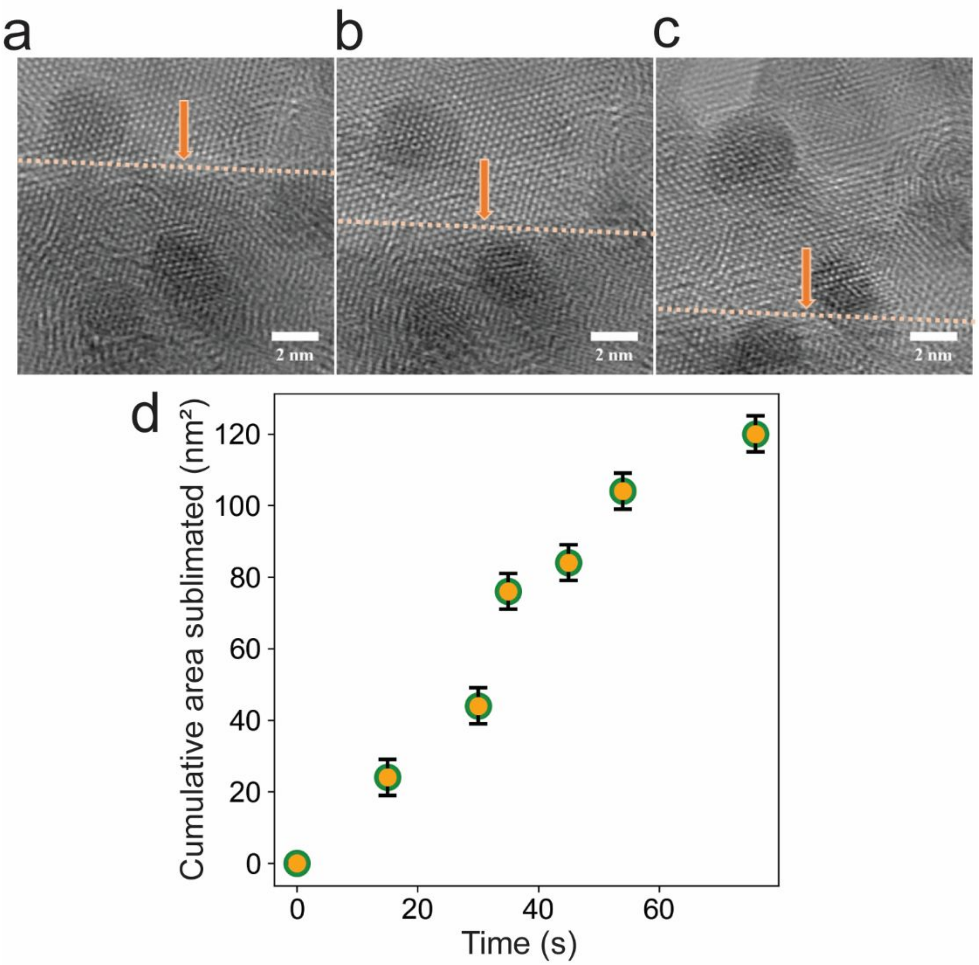

Figure S12. Layer-by-layer sublimation in a few-layered thick Co_9Se_8 NSs at 480 °C. (a-c) Progression of sublimation along {220} crystal facets shown by an arrow. (d) A plot of the cumulative sublimed area as a function of time. (Details on the error bars are given in Supplementary Text 4)

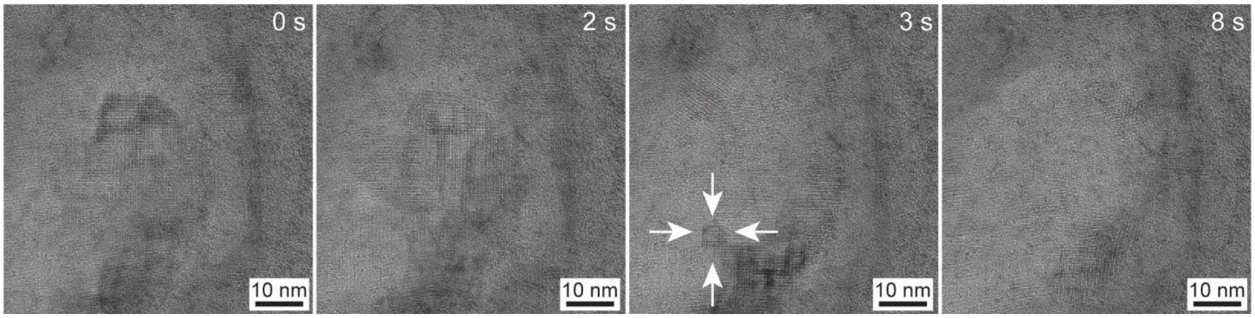

Figure S13. Rapid sublimation in irregularly shaped Co_9Se_8 nanoplate at 520 °C.

Supporting movies

Movie S1: Structural evolution of Co_9Se_8 nanosheet after in situ TEM heating (Sped up X18, frame rate: 150 frames/s).

Movies S2 and S3: Sublimation of Co_9Se_8 NSs oriented in [111] ZA at 500 °C (a) and (b) (Sped up X6, frame rate: 5 frames/s, Sped up X10, frame rate: 5 frames/s)).

Movie S4: Non-continuous and punctuating sublimation of Co_9Se_8 NSs at 460 °C (Sped up X18, frame rate: 25 frames/s).

Movie S5: Semi-continuous sublimation of Co_9Se_8 NSs at 480 °C (Sped up X5, frame rate: 10 frames/s).

Movie S6: Continuous and uniform **s**ublimation of Co_9Se_8 NSs at 500 °C (Sped up X16, frame rate: 10 frames/s).

Movie S7: Layer-by-layer sublimation at 480 °C (Sped up X10, frame rate: 25 frames/s).

Movies S8 and S9: Sublimation in Co_9Se_8 nanobelts at 520 °C (Real time, frame rate: 10 frames/s for both).

Movie S10: Rapid sublimation in Co_9Se_8 nanoplate at 520 °C (Real time, frame rate: 10 frames/s).
